# Alarm with care—a de-implementation strategy to reduce fall prevention alarm use in US hospitals: a study protocol for a hybrid 2 effectiveness-implementation trial

**DOI:** 10.1186/s13012-023-01325-9

**Published:** 2023-12-05

**Authors:** Kea Turner, Molly McNett, Catima Potter, Emily Cramer, Mona Al Taweel, Ronald I. Shorr, Lorraine C. Mion

**Affiliations:** 1 Department of Health Outcomes and Behavior, Moffitt Cancer Center, 12902 Magnolia Drive, Tampa, FL 33612 USA; 2 Department of Gastrointestinal Oncology, Moffitt Cancer Center, 12902 Magnolia Drive, Tampa, FL 33612 USA; 3 Department of Oncological Sciences, University of South Florida Morsani College of Medicine, MFC-EDU, 12902 USF Magnolia Drive, Tampa, FL 33612-9416 USA; 4 Helene Fuld Health Trust National Institute for Evidence-Based Practice, The Ohio State University, 760 Kinnear Road, Columbus, OH 43212 USA; 5 Press Ganey Associates, 1173 Ignition Dr, South Bend, IN 46601 USA; 6 Department of Health Outcomes and Health Services Research, Children’s Mercy Hospital and Clinics, 2401 Gilham Road, Kansas City, MO 64108 USA; 7 Department of Pediatrics, University of Missouri-Kansas City School of Medicine, 2411 Holmes Street, Kansas City, MO 64108 USA; 8 College of Nursing, The Ohio State University, 1577 Neil Avenue, Columbus, OH 43210 USA; 9 Geriatric Research Education and Clinical Center, North Florida/South Georgia Veterans Health System, 1601 SW Archer Road, Gainesville, FL 32608 USA; 10 Department of Epidemiology, College of Public Health and Health Professions, University of Florida, 1225 Center Drive, Gainesville, FL 32611 USA

**Keywords:** Fall prevention, Hospital falls, Inpatient falls, De-implementation, Choosing wisely, Low-value care, Nursing care

## Abstract

**Background:**

Fall prevention alarms are commonly used among US hospitals as a fall prevention strategy despite limited evidence of effectiveness. Further, fall prevention alarms are harmful to healthcare staff (e.g., alarm fatigue) and patients (e.g., sleep disturbance, mobility restriction). There is a need for research to develop and test strategies for reducing use of fall prevention alarms in US hospitals.

**Methods:**

To address this gap, we propose testing the effectiveness and implementation of Alarm with Care, a de-implementation strategy to reduce fall prevention alarm use using a stepped-wedge randomized controlled trial among 30 adult medical or medical surgical units from nonfederal US acute care hospitals. Guided by the Choosing Wisely De-Implementation Framework, we will (1) identify barriers to fall prevention alarm de-implementation and develop tailored de-implementation strategies for each unit and (2) compare the implementation and effectiveness of high- versus low-intensity coaching to support site-specific de-implementation of fall prevention alarms. We will evaluate effectiveness and implementation outcomes and examine the effect of multi-level (e.g., hospital, unit, and patient) factors on effectiveness and implementation. Rate of fall prevention alarm use is the primary outcome. Balancing measures will include fall rates and fall-related injuries. Implementation outcomes will include feasibility, acceptability, appropriateness, and fidelity.

**Discussion:**

Findings from this line of research could be used to support scale-up of fall prevention alarm de-implementation in other healthcare settings. Further, research generated from this proposal will advance the field of de-implementation science by determining the extent to which low-intensity coaching is an effective and feasible de-implementation strategy.

**Trial registration:**

ClinicalTrials.gov identifier: NCT06089239. Date of registration: October 17, 2023.

Contributions to the literature

- This study will advance our understanding of whether coaching is an effective behavior change strategy for de-implementation of low-value care.
- De-implementation has been understudied in nursing care. This study will add to the growing evidence base around how to de-implement low-value nursing care.
- This study is the first that we are aware of that evaluates a de-implementation strategy for reducing fall prevention alarm use in hospitals. Findings from this line of research could be scaled up to additional care settings, given the widespread use of fall prevention alarms in the US healthcare system.

## Background

Falls among hospitalized patients are common, costly, and serious. Each year, up to one million patient falls occur in US hospitals [1]. Patient falls can lead to harm, including injury and death, and increased healthcare costs for patients, payers, and healthcare systems [2–6]. Given the serious consequences, patient falls and fall-related injuries are considered key quality measures for US hospitals. The National Quality Forum recommends that patient falls and fall-related injuries be used as quality measures for nursing-sensitive care [7, 8]. Many healthcare systems monitor total falls and fall-related injuries through reporting systems, such as the National Database of Nursing Quality Indicators® (NDNQI®) [9, 10]. Fall-related injury is also used by the Centers for Medicare and Medicaid Services (CMS) for payment policy. CMS prohibits reimbursement for fall-related injuries during hospitalization [11, 12] and imposes financial penalties for hospitals with poor performance on fall-related injuries [13]. Therefore, hospitals are under significant financial pressure to reduce falls. In response, hospitals have increasingly relied upon unproven strategies, such as fall prevention alarms, since the passage of CMS’s falls-related payment policies [14].

Fall prevention alarms are commonly used among US hospitals as a fall prevention strategy despite limited evidence of effectiveness. A study examining fall prevention practices among 59 acute care nursing units found that over one-third of observed patients (531 out of 1489) had a fall prevention alarm in the “on” position during an audit conducted by nursing staff [15]. Prior studies estimate that 90–99% of US hospitals use fall prevention alarms [16, 17]. While fall prevention alarm use is common, there is limited evidence to support alarm use and evidence that alarms may be harmful. Several randomized controlled trials have demonstrated that fall prevention alarms do not reduce patient falls or fall-related injuries and are not a cost-effective strategy for falls prevention [18, 19]. Fall prevention alarms have a high false-positive rate (ranging from 50 to 99%) and contribute to alarm fatigue among healthcare staff [20–24]. Further, fall prevention alarms lead to mobility restriction, agitation, and sleep disturbance among patients [20, 25–27]. The harm caused by fall prevention alarms has led CMS to restrict the use of fall prevention alarms in nursing homes [25]. Similar efforts are needed to encourage de-implementation of fall prevention alarms in US hospitals.

Few studies have examined barriers to fall prevention alarm de-implementation in US hospitals. Nursing staff often report pressure from hospital leadership to use fall prevention alarms and fear reprimand or punishment if a patient falls during their shift [23, 28]. There is limited research on how to overcome barriers to fall prevention alarm de-implementation, however. To address this gap, we propose testing the effectiveness and implementation of a fall prevention alarm de-implementation strategy, Alarm with Care, using a stepped-wedge cluster-randomized controlled trial (SW-CRT) among 30 medical or medical surgical units from nonfederal US acute care hospitals. Our approach will be guided by the Choosing Wisely De-Implementation Framework [29]. In Aim 1, we will identify barriers to fall prevention alarm de-implementation and develop tailored de-implementation strategies for each unit. In the second aim, we will compare the effectiveness and implementation of high- versus low-intensity coaching to support site-specific de-implementation of fall prevention alarms. We will evaluate implementation and effectiveness outcomes and examine the effect of multi-level (e.g., hospital, unit, and patient) factors on effectiveness and implementation. Fall prevention alarm use is the effectiveness outcome. Change in fall rates including total falls and fall-related injuries will be tracked as the balancing measures. Implementation outcomes will include feasibility, acceptability, appropriateness, and fidelity. Findings from this line of research could be used to support scale-up of fall prevention alarm de-implementation in other care settings. Further, research generated from this proposal will advance the field of de-implementation science by determining whether coaching is an effective and feasible strategy to de-implement low-value care.

## Methods

### Trial design

This hybrid 2 effectiveness-implementation trial will be conducted in two aims. In Aim 1, the study team will employ a mixed-methods approach that includes group concept mapping and focus groups to identify barriers to fall prevention alarm de-implementation and to tailor a core set of de-implementation strategies including education, audit/feedback, and local opinion leaders to each unit. In the second phase, we will use a stepped-wedge cluster-randomized trial (SW-CRT) to compare the effectiveness of high- and low-intensity coaching to de-implement hospital fall prevention alarms using site-specific strategies among 30 medical or medical surgical units.

### Trial registration and funding

This multi-site trial is funded by a grant (R01 AG073408-01A1) from the National Institute on Aging (NIA). This trial has been approved by the Ohio State University Institutional Review Board (study number: 2022B0262). This protocol is reported in accordance with the Standards for Reporting Implementation Studies (StaRI) statement [30] and the SPIRIT guidelines [31]. This trial does meet the NIH definition of a clinical trial and was registered on ClinicalTrials.gov (NCT06089239).

### Data safety and monitoring

A data safety monitoring board of independent experts in the areas of fall prevention, geriatrics, and implementation science will be convened to oversee the data and safety for the current study. The DSMB will review the study’s data safety monitoring plan, manual of procedures, study protocol, and informed consent documents. Further, the DSMB will be responsible for reviewing periodic progress reports on the study (e.g., assessment of data quality, participant recruitment, accrual and retention, and performance at each study site (e.g., intervention adherence)). The DSMB will meet at least every 6 to 9 months. Additionally, the study team will monitor study progress including recruitment, accrual, retention, data quality, and study site performance on an ongoing basis through biweekly meetings.

### Participants

We will target nonfederal acute care hospitals that participate in NDNQI. The following inclusion criteria will be applied: (1) hospitals who have collected, reported, and monitored falls data through the NDNQI managed by Press Ganey for 24 months prior to study enrollment; (2) hospitals who report using fall prevention alarms as a part of their fall prevention protocol; (3) hospitals with an adult medical or medical surgical unit; (4) completion of a signed commitment letter from two senior hospital administrators verifying willingness to participate in the de-implementation intervention; (5) willingness to participate in de-implementation activities (e.g., coaching sessions) and data collection efforts (e.g., fall prevention alarm reporting); and (6) ability to identify a team leader and additional stakeholders to form an interdisciplinary de-implementation team. We will exclude federal hospitals, specialty hospitals (e.g., rehabilitation facilities), and specialty units (e.g., cardiac units), which may have distinct protocols and procedures related to falls prevention compared with general medical or medical surgical units.

### Participant recruitment

We will employ methods used in our prior studies to recruit NDNQI-participating hospitals [15, 17]. First, key partners from Press Ganey will send an invitation to potentially eligible NDNQI hospitals that describes the study purpose, team, and required research activities. Second, we will host webinars at varying days/times to describe the study purpose, team, and required research activities in more detail and answer any questions that potential participants may have. Eligible hospitals that agree to participate will be asked to identify one medical or medical surgical unit to participate.

### Overview of de-implementation strategy

#### Implementation coaching

Coaching is an effective strategy for supporting implementation of evidence-based practices [32, 33]. Coaches often support practice change through additional implementation strategies, such as training and audit and feedback, and can tailor their approach to account for differences in context across settings (e.g., available resources, leadership support) [34]. Coaching is particularly useful for interventions that involve complex change (e.g., change at multiple levels of an organization) and may be well-suited for de-implementation, which may encounter more resistance to change compared with implementation [35].

De-implementation of fall prevention alarms may be affected by several barriers. First, there may be limited awareness that fall prevention alarms are low value given how routinely they are used in hospital settings. Second, the decision to use fall prevention alarms is driven by complex factors (e.g., leadership expectations, fear of reprimand if a patient falls, assessments about patients’ fall risk) [23, 28], requiring deliberate and slow thinking. Habits informed by this type of cognition may be harder to “unlearn” than habits informed by automatic cognition [35]. Third, nurses often work within a hierarchical system and may encounter resistance to change at several levels within hospital settings (e.g., leadership, clinicians from other disciplines). To address these complex barriers, a high-intensity coaching model may be needed that is paired with additional de-implementation strategies including provider education, local opinion leaders, and audit and feedback.

#### Provider education and local opinion leaders

Given that awareness about the evidence for fall prevention alarms may be low, provider education strategies may be a necessary de-implementation strategy. Prior studies suggest that provider education, coupled with other de-implementation strategies, is particularly effective for de-implementing low-value nursing care [36]. Prior studies have demonstrated that nurses report low awareness about low-value care and difficulty with assessing the evidence for an intervention [37, 38], suggesting that provider education may be needed to raise awareness about low-value care. For successful nursing education efforts, prior qualitative studies have recommended strategies, such as designating an individual to oversee education of nursing teams and ensuring that education efforts target vertical key stakeholders (e.g., frontline staff and nursing administration) and horizontal key stakeholders (e.g., nurses, physicians, and other clinicians that play a role in fall prevention efforts) [36, 39]. Local opinion leaders—who are viewed as credible sources of information—can be effective at helping to disseminate information across and within organizations [40]. Therefore, provider education may be most impactful with support from a local de-implementation team and an opinion leader at each site.

#### Audit and feedback

While it is important to increase knowledge about low-value care, education alone may not be sufficient to change behavior. Prior studies have shown that increased knowledge about low-value care does not decrease the use of low-value nursing care [41]. Audit and feedback has demonstrated effectiveness in decreasing the use of low-value care through several behavior change strategies (e.g., setting and monitoring goals) [42, 43]. Prior studies suggest that audit and feedback may be optimized by ensuring that comparisons are made with similar organizations (e.g., similar resource levels) and that the delivery of feedback is individualized and supportive rather than punitive [44]. Further, audit and feedback may be more effective when strategies are taken to help reduce barriers to practice change [45]. Therefore, audit and feedback may work optimally as a strategy paired with health coaches who can provide technical assistance with overcoming site-specific barriers to practice change and who have experience using best practices for delivering feedback to healthcare organizations (e.g., grouping like organizations, providing supportive feedback) [46].

#### Tailoring implementation strategies to each site

The design of each implementation strategy (provider education, local opinion leaders, audit and feedback) may work best if it is tailored to each site. While all sites are likely to encounter a common set of de-implementation barriers (e.g., staff resistance to change), there will likely be site-specific barriers to de-implementation (e.g., hospital protocol encouraging use of fall prevention alarms). Therefore, a tailored approach that considers local barriers at each site may be valuable for de-implementation of fall prevention alarms.

#### Alarm with Care: a multicomponent de-implementation strategy

Alarm with Care combines each of these strategies (coaching, provider education, local opinion leaders, audit and feedback) using a tailored approach to support fall prevention alarm de-implementation. To support tailoring, units will (1) identify and prioritize barriers using group concept mapping and (2) achieve within hospital consensus around targeted barriers and implementation strategies through site-specific focus groups.

During the concept mapping, participating team members for each unit will be asked to identify and prioritize barriers to fall prevention alarm de-implementation using the following prompt, “In order to reduce the use of fall prevention alarms on our unit, we would need to [list suggestions].” The study team will refine the statements (e.g., ensure one idea is captured in each statement, remove irrelevant statements), and unit team members will be asked to group similar statements into piles and rate each statement based on feasibility and importance using a 7-point scale. Concept mapping will be facilitated by the Concept Systems Global Max^©^ GroupWisdom™ secure web-based platform [47].

After concept mapping is complete, one focus group will be scheduled per site. During the focus groups, unit members will discuss barriers to fall prevention alarm de-implementation identified during concept mapping. Sites will review ratings of feasibility and importance for identified barriers and to come to a consensus on which barriers will be prioritized. Sites will review the core set of de-implementation strategies (e.g., provider education, audit and feedback, local champions) and provide feedback on how these strategies may need to be tailored to their site. Sites will also provide feedback on whether additional strategies may be needed for the site (e.g., patient education) based on the prioritized barriers. Sites will be given the flexibility to select additional implementation strategies if needed. Sites will be asked to come to a consensus on which de-implementation strategies will be included at their site. Focus groups approximately 60–90 min in length will be facilitated via videoconference by three study team members (K. T., M. M., L. M.) who have expertise in qualitative methods. Focus groups will be recorded and transcribed verbatim. Data from the concept mapping and focus groups will be used to develop a site-specific de-implementation plan that includes standard operating procedures and a timeline. The plan will be shared with each site, and feedback will be integrated as needed.

Once each site has an established de-implementation plan, hospital units will be randomized to receive either high- or low-intensity coaching services provided by the Helene Fuld National Institute for Evidence-Based Practice in Nursing and Healthcare (i.e., “the Fuld Institute,” located at the Ohio State University College of Nursing). This design was selected to determine whether high-intensity coaching is needed to support successful de-implementation of fall prevention alarms. Coaching sessions will be delivered virtually, and the content will include (1) an introduction to behavior and organizational change theory and principles, (2) implementation facilitation and motivational interviewing for facilitation, (3) building and leveraging teams and opinion leaders, (4) the state of evidence on effective and ineffective fall prevention practices, (5) selection of site-specific de-implementation strategies to address local site barriers, and (6) audit and feedback on fall prevention alarm de-implementation.

Coaching sessions will be led by individuals with a doctorate-level degree who are trained in implementation science. Prior studies have demonstrated the effectiveness of the Fuld Institute coaching services on evidence-based practice implementation including falls prevention [48–50]. Prior to the coaching sessions, all groups will receive educational materials on the state of evidence for effective and ineffective fall prevention practices. While the content of the educational materials will be consistent across sites, the method of distribution of these educational materials will be tailored according to local needs. Once the educational materials have been distributed, coaching sessions will commence.

The amount of coaching will depend upon group assignment. Units randomized to the low-intensity group will receive an initial 2-h orientation session and access to monthly office hours for discussion about group progress and troubleshooting barriers. Units randomized to the high-intensity group will receive an initial 4-h orientation, weekly coaching sessions for the first month of implementation, and monthly coaching sessions thereafter, access to office hours, and access to “on call” assistance to troubleshoot problems in real time. Both groups will receive instruction on use of the Fuld Institute Implementation for Sustainability Toolkit [51], which allows sites to create a tailored implementation plan and timeline for de-implementation.

### Randomization

The randomization scheme for this SW-CRT is presented in Table 1. Recruitment will be stratified by hospital size (< 300 beds vs. ≥ 300 beds) and teaching status (e.g., teaching vs. non-teaching). Hospitals within each of the four strata will be randomly selected from the hospitals that sign up to participate. Within each stratum, hospitals will be matched based on fall prevention alarm use, and matched pairs will be randomly assigned using a random allocation sequence to one of three waves and to either the high- or low-intensity coaching condition. All sites will be identified and recruited prior to randomization. The allocation sequence will be concealed until all clusters are enrolled and assigned to the intervention. This design was chosen to ensure equivalence across conditions based on baseline fall prevention alarm use and organizational characteristics that may affect resources available for de-implementation efforts (e.g., hospital size, teaching status). Due to the nature of the intervention, it is not possible to blind study participants or interventionists (e.g., coach). Outcome assessors will be blinded to units’ assigned study condition.

**Table 1:** Randomization scheme for stepped-wedge cluster-randomized controlled trial, *N* = 30 hospital units

**Study Year**	**Year 2**				**Year 3**				**Year 4**			
**Quarter**	**Q1**	**Q2**	**Q3**	**Q4**	**Q5**	**Q6**	**Q7**	**Q8**	**Q9**	**Q10**	**Q11**	**Q12**
**Wave 1** High Intensity (*n* = 5)	P	I	S	S	S	S	S	S				
Low Intensity (*n* = 5)	P	I	S	S	S	S	S	S				
**Wave 2** High Intensity (*n* = 5)		P	I	S	S	S	S	S	S			
Low intensity (*n* = 5)		P	I	S	S	S	S	S	S			
**Wave 3** High intensity (*n* = 5)			P	I	S	S	S	S	S	S		
Low intensity (*n* = 5)			P	I	S	S	S	S	S	S		

P: Planning phase will include site-specific focus groups and development of tailored implementation strategies

I: Implementation phase will include receipt of coaching intervention and tailored implementation strategies

S: Sustainability phase will include transition of implementation activities to local site and data monitoring

### Data collection and measures

#### Effectiveness outcome

The effectiveness outcome will be measured by assessing monthly prevalence of fall prevention alarm use using an investigator-developed observation form. The form collects information on number of observed patients and number of observed patients with the alarm position in the “on” position. Observations will be conducted by a nurse data collector, a method that has been used in our prior studies [15, 17, 52].

#### Balancing measures

We will also measure change in total patient fall rates and fall-related injuries using NDNQI measures, which have been previously validated [53, 54]. Fall rates and fall-related injuries are reported as number of incidents per 1000 patient days. Data on NDNQI measures will be obtained from the NDNQI database managed by Press Ganey. Fall rates and fall-related injuries will be reported at the hospital unit level.

#### Implementation outcomes

Implementation outcomes will be measured by assessing staff perceptions about feasibility, acceptability, and appropriateness of the de-implementation *process* (e.g., implementation coaching and other de-implementation strategies) using previously validated measures [55]. Additionally, we will measure staff perceptions about the acceptability and appropriateness of the practice (i.e., staff perceptions about whether it is agreeable to reduce fall prevention alarm use) using similar measures. Frontline workers responsible for fall prevention (e.g., nurse) and organizational leaders responsible for setting expectations and providing support for falls prevention (e.g., director of nursing) will be surveyed. Scores will be aggregated at the hospital level. Fidelity will be measured using a checklist to document which components of the implementation plan are delivered at each site to ensure consistency across coaching sessions. Intervention adherence will be measured as the percentage of coaching sessions attended and adherence to the implementation plan (e.g., percentage of core de-implementation strategies completed by each site). Intervention quality will be measured using study team developed items that assess satisfaction with coaching services (e.g., content, delivery, facilitator, scheduling/timing).

#### Additional measures

Data from the NDNQI database and clinician-level surveys will be used to capture multi-level factors that may influence the effectiveness and/or implementation of the de-implementation strategy. *Patient-level* data will include socio-demographics. *Unit-level* data will include unit type (e.g., medical or medical-surgical), staffing characteristics (e.g., staffing ratios), and use of fall prevention interventions that may affect fall alarm prevention use (e.g., availability of companions/sitters). *Hospital-level* data will include hospital characteristics that may affect fall prevention alarm use (e.g., hospital size, teaching status, rural location).

### Sample size

The primary outcome of interest in this study is reduction in fall prevention alarm use. To determine an adequate sample size for estimating a clinically significant difference in alarm use, we conducted a simulation study. We defined a 10% reduction in fall prevention alarm use from baseline in the low-intensity coaching condition and a 15% reduction in fall prevention alarm use in the high-intensity coaching condition as a clinically significance difference. Based on our prior studies, the following assumptions were made for the simulation study: (1) an intra-unit correlation of 0.5, (2) an average of 24 patients assessed per unit each month, and (3) a 33% baseline rate of alarm use. Using a mixed logistic regression model, odds of alarm use in a unit month were determined by study condition (low intensity, high intensity) and a random unit intercept. After calculating each unit’s probability of alarm use, we conducted a random binomial draw for the number of alarms in use, assuming a 20% attrition rate. Power was defined as the proportion of datasets with *P* < 0.05 for the intervention effect test. The required sample size for the study was determined 30 units (26 units after attrition). The power to detect a 10% difference for the low-intensity condition was 96%.

### Data analysis plan

Study results will be presented in accordance with the CONSORT extension for SW-CRT [56]. We will use an intent-to-treat analytic approach. We will compare participant hospital characteristics with nonparticipating NDNQI hospitals to examine for potential selection bias. We will compare hospitals who withdrew from the study early to those who completed the study to examine for potential attrition bias. We will use multiple imputation methods to address missing data if necessary.

Odds of alarm use will be modeled as a function of study condition using a generalized logistic mixed model with a random unit intercept to account for potential clustering. A similar approach will be used for comparing implementation outcomes across study conditions. Additional models will be estimated to identify potential correlates of fall prevention alarm use (e.g., patient, unit, hospital characteristics). For example, implementation outcomes (e.g., unit adherence to de-implementation strategy) may be associated with fall prevention alarm use.

The data analysis plan will also include mixed-methods data collection including group concept mapping and focus groups. For the sorted statements generated through the concept mapping, we will create a similarity matrix to quantify the similarity between every possible pair of statements and develop a point map to visually display the similarity between statements. Additional visual displays, such as a cluster rating maps and pattern matches, will be developed to visually display average scores on feasibility and importance ratings and to compare average scores across groups (e.g., nurses versus physicians’ rating of importance). We will use a hybrid approach of integrating inductive and deductive coding to analyze focus group data, an approach commonly used in implementation science qualitative methods [57, 58]. We will develop deductive codes based on topics identified in the focus group guide [59] and develop additional inductive codes based on themes that emerge from the data [60]. Independent coders will code the data and come to a consensus regarding code application.

## Discussion

The overall goal of this study is to examine the effectiveness and implementation of Alarm with Care, a de-implementation strategy to reduce the use of fall prevention alarms in US hospitals. While fall prevention alarms are widely used, there is no evidence to demonstrate their effectiveness and studies to suggest alarms may be harmful to healthcare staff and patients [18, 19]. There has been limited study of barriers to fall alarm de-implementation or the development and testing of strategies to reduce fall prevention alarm use. The Alarm with Care strategy seeks to overcome this research gap by testing a first-in-kind fall prevention alarm de-implementation initiative in US hospitals. Findings from this line of research could be scaled up to additional healthcare settings where fall prevention alarms are used (e.g., specialty hospitals). Further, information from the study will contribute to the growing literature on de-implementation science by determining whether coaching, paired with provider education and feedback, is an effective strategy for reducing low-value care.

## Data Availability

Due to collection of sensitive, protected health information, data from this study cannot be made publicly available.

## Abbreviations

CMS: Centers for Medicare and Medicaid Services
DSMB: Data Safety Monitoring Board
NDNQI: National Database of Nursing Quality Indicators
NIA: National Institute on Aging
NIH: National Institutes of Health
SW-CRT: Stepped-wedge cluster-randomized trial
US: United States
